# Adsorption of 1,2-Dichlorobenzene from the Aqueous Phase onto Activated Carbons and Modified Carbon Nanotubes

**DOI:** 10.3390/ijms222313152

**Published:** 2021-12-05

**Authors:** Martyna Jurkiewicz, Robert Pełech

**Affiliations:** Department of Chemical Organic Technology and Polymeric Materials, Faculty of Chemical Technology and Engineering, West Pomeranian University of Technology in Szczecin, 70-322 Szczecin, Poland; robert.pelech@zut.edu.pl

**Keywords:** 1,2-dichlorobenzene, adsorption, activated carbon, modified carbon nanotubes

## Abstract

This study aimed to describe the adsorption process of ortho-dichlorobenzene (o-DCB) onto activated carbons (ACs) and modified carbon nanotubes (CNTs) from the aqueous phase. The starting material NC_7000 carbon nanotubes were modified by chlorination (NC_C) and then by the introduction of hydroxyl groups (NC_C_B). The concentration of o-DCB in solutions was performed by UV-VIS spectrophotometry. After adsorption, the activated carbons were regenerated by extraction with organic solvents such as acetone, methanol, ethanol, and 1-propanol; the carbon nanotubes were regenerated by methanol. The degree of adsorbate recovery was determined by gas chromatography (GC) with flame ionization detection, using ethylbenzene as an internal standard. The equilibrium isotherm data of adsorption were satisfactorily fitted by the Langmuir equations. The results indicate that carbon adsorbents are effective porous materials for removing o-DCB from the aqueous phase. Additionally, activated carbons are more regenerative adsorbents than carbon nanotubes. The recoveries of o-DCB from ACs were in the range of 76–85%, whereas the recoveries from CNTs were in the range of 23–46%. Modifications of CNTs affect the improvement of their adsorption properties towards o-DCB compared to unmodified CNTs. However, the introduction of new functional groups on carbon nanotube surfaces makes the regeneration process less effective.

## 1. Introduction

Compared with other abatement methods, the adsorption process has been recognized as the most effective, especially with carbon materials as an adsorbent. Adsorption can be defined as an increase in the concentration of a substance (adsorbate) on a surface (adsorbent). Then adsorbate forms a molecular film on the adsorbent’s surface. In addition, it is a non-destructive technique [1,2]. Throughout adsorption, various types of organic compounds are removed from aqueous solutions, e.g., chloroderivatives, such as 1,2—dichlorobenzene, dyes such as methylene blue, and numerous volatile organic compounds [3,4,5,6].

Activated carbon is a microporous adsorbent that is characterized by significant porosity and developed surface area. Their physical and sorption properties depend on the raw material, the method of preparation and activation, as well as the modification of the surface groups [7]. Due to their wide range of properties, activated carbons are ideal for removing substances such as carbon dioxide [8], volatile organic compounds [9], or inorganic ions like copper, zinc, and chromium from a gas or aqueous phase [10]. Carbon nanotubes (CNTs) are carbonaceous materials that have been successfully used to remove pollutants from the aqueous and gaseous phases. Many investigations show that nanotubes are effective adsorbents for removing fluoride [11], dioxin [12], or lead [13]. 

**Figure 1:**
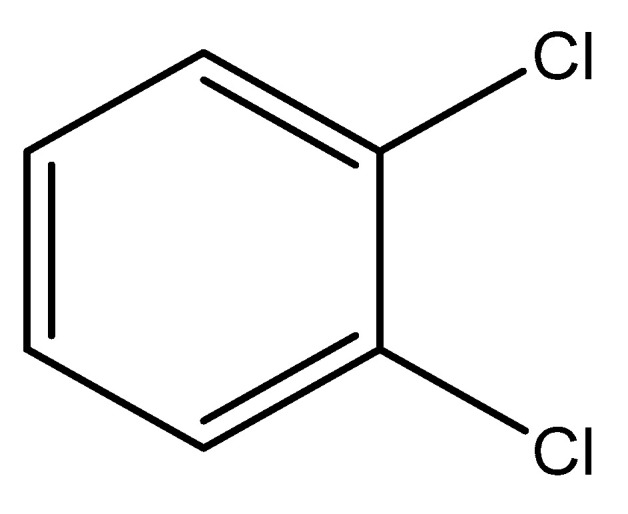
Chemical formula of 1,2-dichlorobenzene.

Dichlorobenzene (DCB) is a benzene derivative. It is a co-product of a benzene chlorination reaction by electrophilic substitution mechanisms in the presence of iron (III) chloride as a catalyst. Dichlorobenzenes are toxic and harmful to organisms. Dichlorobenzenes have found use in the synthesis of dyes, insecticides, paint, and coat solvents [14]. o-DCB is a colorless liquid with a characteristic odor. It is used as a solvent, an intermediate product in the synthesis of 3,4-dichloroaniline, a component of insecticidal preparations and deodorants [15]. 1,2-dichlorobenzene goes into the water through the chemical discharge of industrial plants. It has the ability to accumulate in animal tissues and change the taste of water [16]. 1,2-DCB is slightly soluble in water. Therefore, adsorption is an appropriate way to remove 1,2-DCB from water as it is present in low concentrations [17]. 1,2-DCB is more soluble in methanol, ethanol, or benzene. Many studies on the adsorption of chlorinated compounds have shown that methanol does not influence the adsorption process [18,19]. The structure of the o-DCB molecule is shown in Figure 1.

Chen et al. investigated the adsorption of 1,2-DCB onto Dickinson natural sediment. 1,2-dichlorobenzene solutions were prepared in methanol and then in the electrolyte solution (NaCl, CaCl_2_, NaN_3_). For this sorbate, the maximum adsorption capacity was 6.37 µg/g [20]. Peng et al. published a study on the adsorption of 1,2-DCB onto as-grown and graphitized carbon nanotubes from an aqueous solution. The time to reach equilibrium was 40 min for both adsorbents, at an initial concentration of 20 mg/g. The adsorption capacity on as-grown nanotubes was 30.8 mg/g, while that on graphitized nanotubes was 28.7 mg/g. Their results indicated that carbon nanotubes are effective adsorbents in the removal of 1,2-DCB at pH values ranging from 3 to 10 [17]. 1,2-dichlorobenzene can be successfully removed from the aqueous solution using flat and stepped Au and Pt surfaces. In this case, adsorption is possible due to dispersion interactions [21]. Deitsch et al. performed adsorption of 1,2-DCB on peat soil and organobentonites. The study showed that for this adsorbate, organobentonites were the more efficient adsorbent. It was also found that the length of the organobentonite alkyl chain did not affect the adsorption rate. However, the longer the alkyl chain, the lower the desorption rate. The adsorption equilibrium for both adsorbents was linear over the concentration ranges studied [22]. A separate study of the adsorption of 1,2-DCB on natural sorbents indicated that the equilibrium of the process is well described by the Freundlich model [23]. Bullot et al. described adsorption of 1,2-DCB onto MIL-101 (Cr) nano- and microcrystals. They found that adsorbate diffusion is faster for microcrystals than for nanocrystals. The maximum adsorption capacity was 1670 mg/g [24].

## 2. Results and Discussion

### 2.1. Equilibrium of o-DCB Adsorption

The Langmuir adsorption isotherm equation (Equation (1)) was used to describe the equilibrium of o—DCB adsorption onto activated carbons and carbon nanotubes. To determine the values of *a_m_* and *b*, the equation was reduced to its linear form C/a = f(C).

(1) $$a=\frac{a_{m}\cdot b\cdot C}{1+b\cdot C}$$

where the constant *a_m_* is the adsorbent capacity (maximum adsorption amount) expressed in mg/g, and *b* is the Langmuir equilibrium constant. 

Coefficient values of the Langmuir adsorption model shown in Table 1 indicate that AG-5 AC has a higher monolayer adsorption capacity than DT0 AC. Modification of CNTs increased the *a_m_* coefficient by 16% (NC_C) and 35% (NC_C_B) compared to NC_7000. 

**Figure 2:**
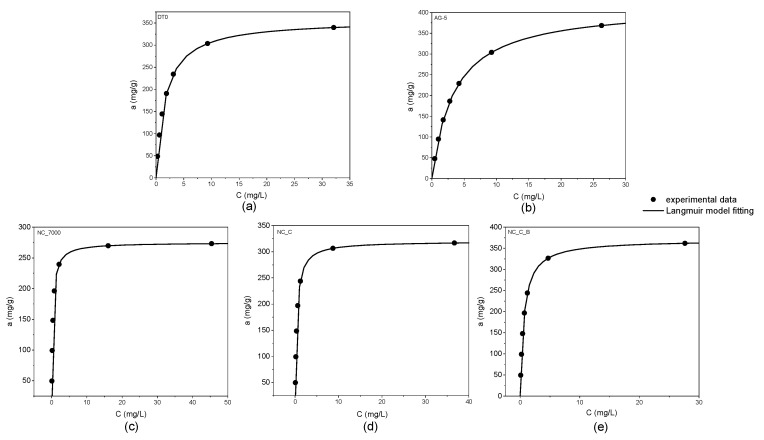
Adsorption isotherms of o-DCB onto DT0 (**a**), AG-5 (**b**), NC_7000 (**c**), NC_C (**d**), and NC_C_B (**e**).

According to the IUPAC classification, the adsorption isotherms shown in Figure 2 are of I type. The graphs below demonstrate the excellent agreement of the experimental data with the Langmuir equation (R^2^ > 0.99).

**Figure 3:**
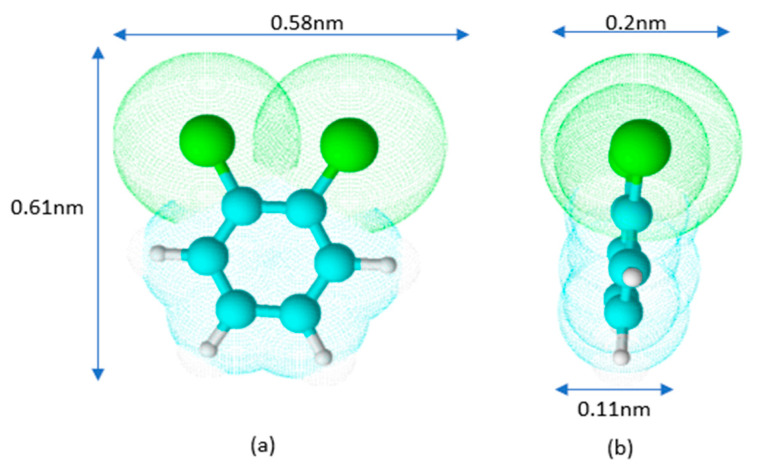
Model of o-DCB molecule in front (**a**) and side (**b**) views.

Schematically, the shape of the o-DCB molecule is represented in Figure 3.

The estimation of dimensions was based on the bond lengths of C-C 0.14 nm, C-H 0.11 nm, and C-Cl 0.174 nm. From the above assumption, it follows that depending on the orientation of the molecule on the surface, its cross-sectional area (A) can vary from approximately 0.06 (0.11 × 0.58) to 0.35 (0.58 × 0.61) nm^2^.

The degree of surface coverage S_c_ for *a_m_* values summarized in Table 2 indicates that the adsorption of o-DCB on CNTs occurs with the orientation of the DCB molecules. The values calculated for activated carbons show that o-DCB molecules form a monomolecular layer on their surface.

(2) $$a_{m1}=\frac{a_{m}}{M}$$

(3) $$a_{m2}=\frac{a_{m}}{M\cdot S_{BET}}\cdot N$$

(4) $$S_{c1}=a_{m2}\cdot A_{1}$$

(5) $$S_{c2}=a_{m2}\cdot A_{2}$$

**Figure 4:**
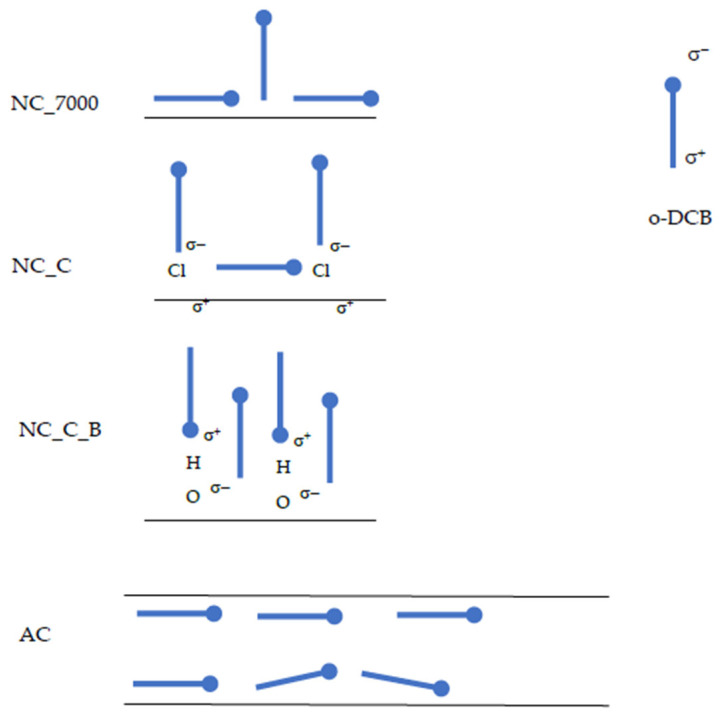
Scheme of the orientation of o-DCB molecules on the surface of AC and CNTs. The black lines represent the surface of the adsorbent—for CNTs, the outer layer of the adsorbent, for ACs, the inner surface of the pore.

We suppose that o-DCB molecules are placed horizontally on the surface of activated carbons. In the case of carbon nanotubes, higher values of S_c_ indicate that a monolayer may be formed, with some particles arranged vertically and some horizontally. This phenomenon may be due to the microporosity of the activated carbons. In pores smaller than 1.5 nm, the particles cannot orient themselves vertically. In contrast, o-DCB adsorbs on the outer surface of carbon nanotubes. This enables easier vertical orientation of the adsorbate molecules. Moreover, the modification of the carbon nanotube surfaces enhances this effect. The chlorine atoms and hydroxyl groups introduced on the CNT surfaces cause polarization on the surface. The presence of strongly electronegative chlorine atoms in the o-DCB molecule also cause its polarization, forming a dipole. Graphically, this process is shown in Figure 4.

We find that as the hydrophilicity of the adsorbent surface increases, the constant *b* of the Langmuir equation decreases. The constant *b* in this equation reflects the equilibrium state. For a more hydrophilic surface, the competition of water (solvent) with o-dichlorobenzene for sorption sites increases. This, in turn, moves some of the o-DCB molecules into solution. This is similar during adsorption on activated carbons, which are obtained by steam-gas activation. Then, oxygen groups are introduced on the surface of the adsorbent. Hence, the significantly lower values of the *b* constant compared to more graphitized CNTs. 

### 2.2. Kinetics of Adsorption of o-DCB onto ACs and CNTs

It was found that the kinetic constants of adsorption onto carbon nanotubes do not depend on the degree of coverage of the adsorbent. For adsorption onto activated carbons, the adsorption rate constant decreases as the degree of coverage increases. Values of kinetic constants are listed in Table 3. 

**Figure 5:**
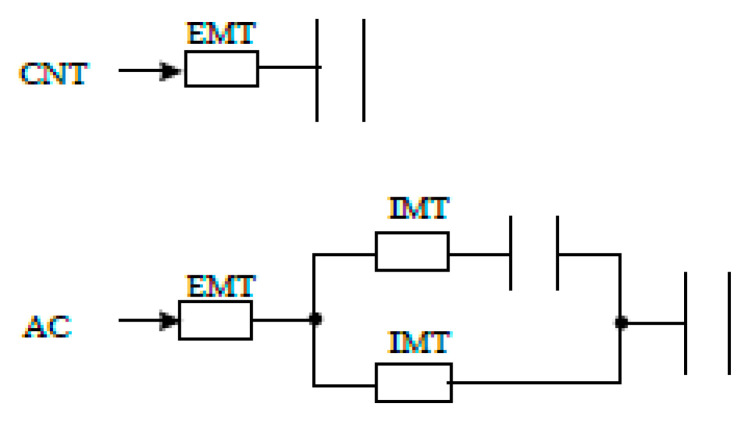
Scheme mass transfer of o-DCB to CNTs and ACs surfaces. EMT, external mass transfer; IMT, internal mass transfer. The resistor symbol represents the mass transfer resistance, and the capacitor symbol the adsorption capacity of the adsorbent.

In a simplified way, it can be assumed that the adsorption of o-DCB onto CNTs and ACs occurs as described in Figure 5.

The adsorption of o-DCB followed a pseudo-first-order kinetic model, which is described by:

(6) $$\frac{dC}{dt}=k\cdot \left(C_{n}-C_{e}\right)$$

(7) $$ln\left(\frac{C_{0}-C_{e}}{C_{n}-C_{e}}\right)=k\cdot C+B$$

where *C*_0_ is the o-DCB initial concentration, *C_e_* is the o-DCB equilibrium concentration, *C_n_* is the concentration of o-DCB in time *t* for the first run B = 0. 

**Figure 6:**
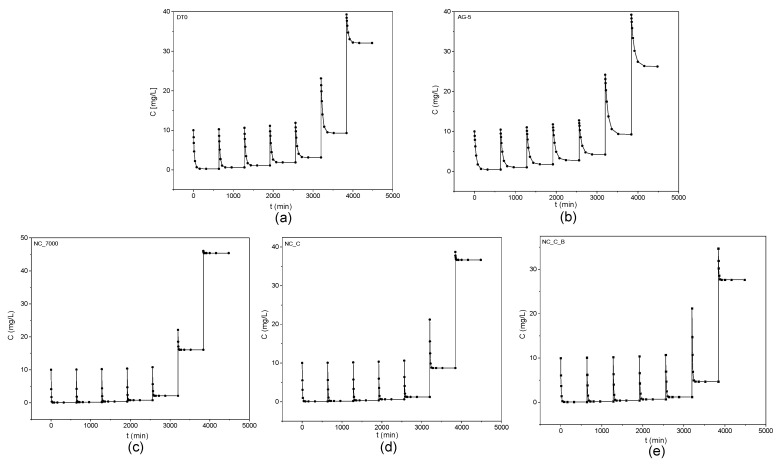
Kinetic curves of o-DCB adsorption onto DT0 (**a**), AG-5 (**b**), NC_7000 (**c**), NC_C (**d**), and NC_C_B (**e**).

**Figure 7:**
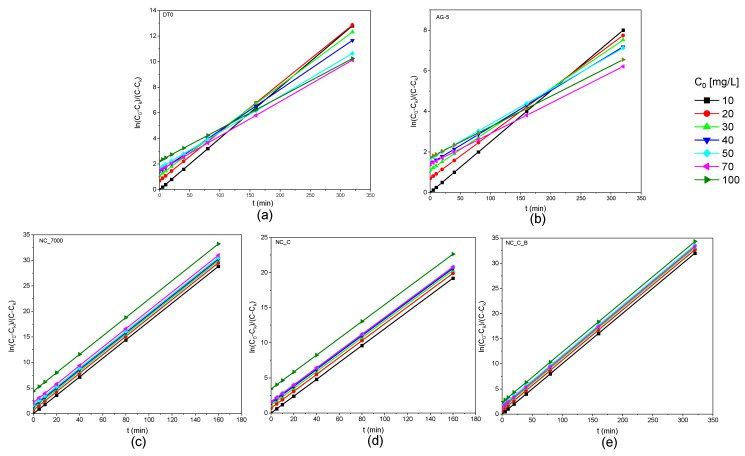
Kinetic curves of o-DCB adsorption onto DT0 (**a**), AG-5 (**b**), NC_7000 (**c**), NC_C (**d**), and NC_C_B (**e**) in linear form.

Figure 6 shows the obtained kinetic curves of o-DCB adsorption on the tested adsorbents and Figure 7 shows the kinetic curves in a linearized system.

### 2.3. Regeneration of Adsorbents

The highest recovery of o-DCB from activated carbons was obtained using methanol as a solvent. As expected, the recovery of o-DCB from carbon nanotubes was significantly lower than for activated carbons. Additionally, in the case of modified carbon nanotubes, the presence of chlorine and hydroxyl functional groups hindered the regeneration of the adsorbents. The modifications of CNT caused larger interactions of the introduced groups with o-DCB so that the adsorbent was less regenerated. Dipoles interact more with the CNT surface. Detailed results of the regeneration of adsorbents are presented in Table 4. The higher regeneration rate of activated carbons indicates o-DCB molecules have a lower affinity for the surface of ACs than for CNTs. This is also confirmed by the constant *b* of the Langmuir equation.

## 3. Materials and Methods

### 3.1. Adsorbents

Commercial DT0 and AG-5 (Grand Activated Sp. z o.o.) activated carbons, NC_7000 (Nanocyl), modified NC_C, and NC_C_B carbon nanotubes were used as adsorbents. Details of the synthesis and characterization of the adsorbents used are described in the previous works [25,26]. The specific surface areas of adsorbents are presented in Table 5. The adsorbate was 1,2-dichlorobenzene (anhydrous, 99%, MERCK). 

**Figure 8:**
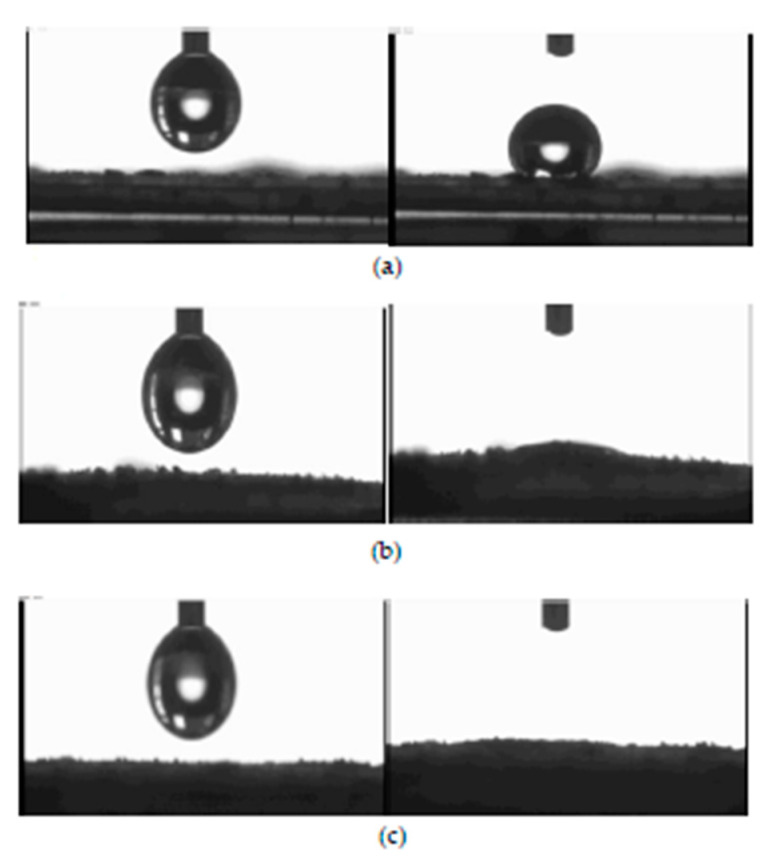
Wetting properties of using NC_7000 (**a**), NC_C (**b**), and NC_C_B (**c**) CNTs.

The wettability test was carried out using an Automated Melting Point System OptiMelt goniometer. For this purpose, tablets with a 1 cm diameter were pressed from 200 mg CNTs samples. The tablets were made using a hydraulic press at a pressure of 10 tones. Water droplet images on CNT surfaces are presented in Figure 8.

Unmodified NC_7000 carbon nanotubes were found to have a strongly hydrophobic surface. In contrast, NC_C and NC_C_B samples are very easily wetted, and their surface is, therefore, more hydrophilic.

### 3.2. Adsorption Procedure

Solutions of o-DCB at concentrations of 1, 2, 5, 8, and 10 mg/L were prepared in 500 cm^3^ Erlenmeyer flasks. The solutions were formed by diluting a stock solution of o-DCB in methanol at a concentration of 50 g/L with distilled water. The flasks were closed with a stopper and mixed thoroughly. The absorbance of each was determined three times in a quartz cuvette at 197 nm by spectrometry using a Spectroquant Pharo 300 apparatus (MERCK). A graph of the concentration dependence of absorbance was plotted. Next, in 500 cm^3^ flat bottomed flasks, 500 cm^3^ of distilled water and an appropriate volume (0.1 mL) of stock solution were placed to give an initial concentration of 10 mg/L. Then, a weighted adsorbent (100 mg) was poured in and stirred with a magnetic stirrer. After 5, 10, 20, 40, 80, 160, 320, and 640 min, the absorbance of the solution was measured, and another portion of the stock solution (0.1 mL) was added. This was repeated five more times. In further steps, 0.2 and 0.3 mL were added, which gives the total initial concentration of 100 mg/L. The adsorption capacity was calculated using the following formula:

(8) $$a=\frac{\left(C_{0}-C_{e}\right)\cdot V}{m}\;[\mathrm{mg}/g]$$

where *C*_0_ and *C_e_* are the initial and equilibrium concentrations, respectively [mg/L]. V is the volume of solution [L], and m is the weight of an adsorbent [mg].

In order to regenerate the activated carbons, the contents of the flasks were drained under reduced pressure, and the carbons on filters were placed in 100 cm^3^ screw-top jars. Into each was poured 40 mL of 1-acetone, 2-ethanol, 3-methanol, and 4-1-propanol, and then they were left on a TS–2 Orbital Shaker set at 130 rpm for 48 h. For the regeneration of carbon nanotubes, methanol was used as an extractant. The amount of o-DCB extracted from the adsorbents was determined by gas chromatography using the standard internal method, which was ethylbenzene. The study was performed using a Thermo Electron GC 8000 Gas Chromatograph with FID (flame ionization detector). Chromatographic analysis conditions:

− Hydrogen flow rate: 35 mL/min;
− Air flow rate: 350 mL/min;
− Analysis temperature: 240 °C;
− Temperature increment: 15 °C/min;
− Time per analysis: 25.33 min;
− Partition coefficient: 15.

In a 25 cm^3^ flask, a solution of o-DCB in acetone was prepared at the maximum concentration to be expected from the extraction. The internal standard was added to the flask in portions of 20, 40, 60, 80, 100 µL, successively. After each addition of internal standard, the flask contents were stirred, and 0.2 µL were injected twice. Then 40 µL of the internal standard was added to each jar after shaking and mixed thoroughly. Two injections of 0.2 µL of each solution were made from the adsorbents, and the volume of o—dichlorobenzene in the samples was calculated from the calibration curve equation. The volume (V) was converted to mass (*m_o-DCB_*) and divided by the amount of adsorbed o-DCB [mg] (*a*_0_) to calculate the recovery (%*R*) of the adsorbate:

(9) $$\%R=\frac{m_{o-DCB}}{a_{0}}\cdot 100\%$$

## 4. Conclusions

In the present study, we have examined the adsorbability of o-DCB onto activated carbons and carbon nanotubes from an aqueous solution. AG-5 activated carbon is the most regenerable, and it has the best adsorption affinity towards o-DCB. Our study shows that activated carbons are a better choice for the adsorption of o-DCB from an aqueous solution than carbon nanotubes. Activated carbons also have an economic advantage over carbon nanotubes—they are cheaper and more readily available. The Langmuir equation shows a satisfactory fit of the o-DCB adsorption onto carbon adsorbent. The regeneration by extraction of activated carbons is most effective with methanol as a solvent.

Surface modification of carbon nanotubes by introducing chlorine and hydroxyl groups improves their adsorption properties towards o-DCB compared to unmodified carbon nanotubes. The recovery rate of adsorbate from carbon nanotubes is lower than from activated carbons. What is more, modifications of carbon nanotubes reduce the recovery rate of o-DCB.

Based on these results, we conclude that activated carbons and modified carbon nanotubes are promising adsorbents for o-DCB removal from aqueous solution. 

## Figures and Tables

**Table 1:** Coefficient values of the Langmuir adsorption isotherm equation.

Adsorbent	Coefficients of Langmuir Equation	
	*a_m_*	*b*
DT0	357	0.61
AG-5	417	0.29
NC_7000	275	3.2
NC_C	320	2.6
NC_C_B	370	1.7

**Table 2:** Surface coverage of adsorbents for different cross-sectional areas.

Adsorbent	*a_m_*_1_ [mmol/g]	*a_m_*_2_ Amount of o-DCB per nm^2^ [molecules/nm^2^]	S_c1_ for *A*_1_ = 0.35 nm^2^	S_c2_ for *A*_2_ = 0.06 nm^2^
DT0	2.4	1.54	0.55	0.09
AG-5	2.8	1.99	0.72	0.12
NC_7000	1.9	6.16	2.22	0.37
NC_C	2.2	7.12	2.56	0.43
NC_C_B	2.5	9.13	3.29	0.55

**Table 3:** Values of kinetic constants at different initial concentrations.

Adsorbent	Kinetic Constants *k* at Initial Concentration *C*_0_ [mg/L]						
	10	20	30	40	50	70	100
DT0	0.040	0.038	0.035	0.032	0.028	0.027	0.025
AG-5	0.025	0.022	0.020	0.018	0.017	0.015	0.015
NC_7000	0.180	0.180	0.180	0.180	0.180	0.180	0.180
NC_C	0.120	0.120	0.120	0.120	0.120	0.120	0.120
NC_C_B	0.100	0.100	0.100	0.100	0.100	0.100	0.100

**Table 4:** Comparison of recovery of o-DCB from ACs and CNTs.

Solvent	Recovery of o-DCB R [%] from				
	DTO	AG-5	NC_7000	NC_C	NC_C_B
acetone	76	79			
methanol	80	85	46	23	26
ethanol	78	75			
1-propanol	77	80			

**Table 5:** Specific surface areas of adsorbents.

Adsorbent	S_BET_ [m^2^/g]
DT0	950
AG-5	860
NC_7000	183
NC_C	184
NC_C_B	166

## Data Availability

All data generated in this study is presented in the current manuscript. Data is available upon request from the corresponding author.
